# Determining Preparation-Induced Differences in Local Forces Acting along Polymer Chains in Spin-Coated Films

**DOI:** 10.1021/acsmacrolett.6c00311

**Published:** 2026-07-24

**Authors:** Meirui Fu, Raphael Hertel, Bence Dajka, Michael Walter, Michael Sommer, Günter Reiter

**Affiliations:** † Institute of Physics, University of Freiburg, Freiburg 79104, Germany; ‡ Institute for Chemistry, Chemnitz University of Technology, Chemnitz 09111, Germany; § FIT Freiburg Centre for Interactive Materials and Bioinspired Technologies, University of Freiburg, Freiburg 79110, Germany

## Abstract

We employed photoluminescence (PL) from mechanochromophores covalently linked into the backbone of a glassy polymer as optical force sensors (“optical springs”) to resolve forces acting along polymer chains in spin-coated thin films. From the shift of the peak position of the PL spectrum, we deduced changes in the main force. Changes in the shape of the spectra were interpreted as alterations in populations of polymer chains experiencing lower and higher forces than the main force. Depending on the conditions of spin coating, expressed through a preparation parameter *p*_ev_, we observed distinct changes in the corresponding PL spectra of the resulting polymer films, reflecting characteristic variations in the distribution of forces acting along polymer chains as a function of *p*_ev_. Our results allowed to link the forces acting along individual polymer chains in spin-coated polymer films with macroscopically detected residual stresses, with the latter reflecting the combined and cooperative response of a large ensemble of polymer chains. Interestingly, residual stresses and the forces acting along individual polymer chains did not exhibit the same dependence on *p*_ev_.

## Graphical abstract

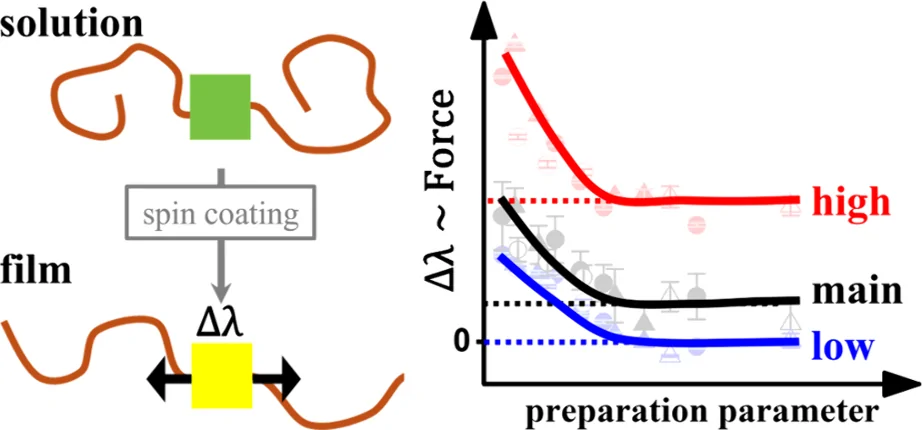

Thin polymer films are frequently an integral part of modern technologies, serving as protective barriers,^1,2^ dielectric layers in electronics,^3,4^ and precision optical coatings.^5,6^ Such films are often fabricated via spin coating, a highly reproducible solution-based casting technique that enables the rapid production of uniform thin layers.^7,8^ During spin coating, solvent evaporation continuously increases the polymer concentration in the spreading solution film, progressively slowing chain relaxation.^9,10^ Eventually, solvent removal becomes faster than polymer equilibration, and the film thus enters a vitrification regime with strongly suppressed segmental mobility. Beyond this point, polymer chains can no longer equilibrate, even though a significant amount of solvent is still present in the film.^11,12^ Because the lateral dimensions of the polymer film are fixed by the substrate, continued loss of solvent leads to a contraction of the film primarily in the thickness direction, such that nonequilibrated polymer chains are frozen in the dry film (Figure 1a). Thus, arising from nonequilibrated chain conformations, residual stresses are present in the spin-coated film with properties mainly controlled by the initial polymer concentration *c* of the solution and the spin speed ω.^13,14^

**Figure 1:**
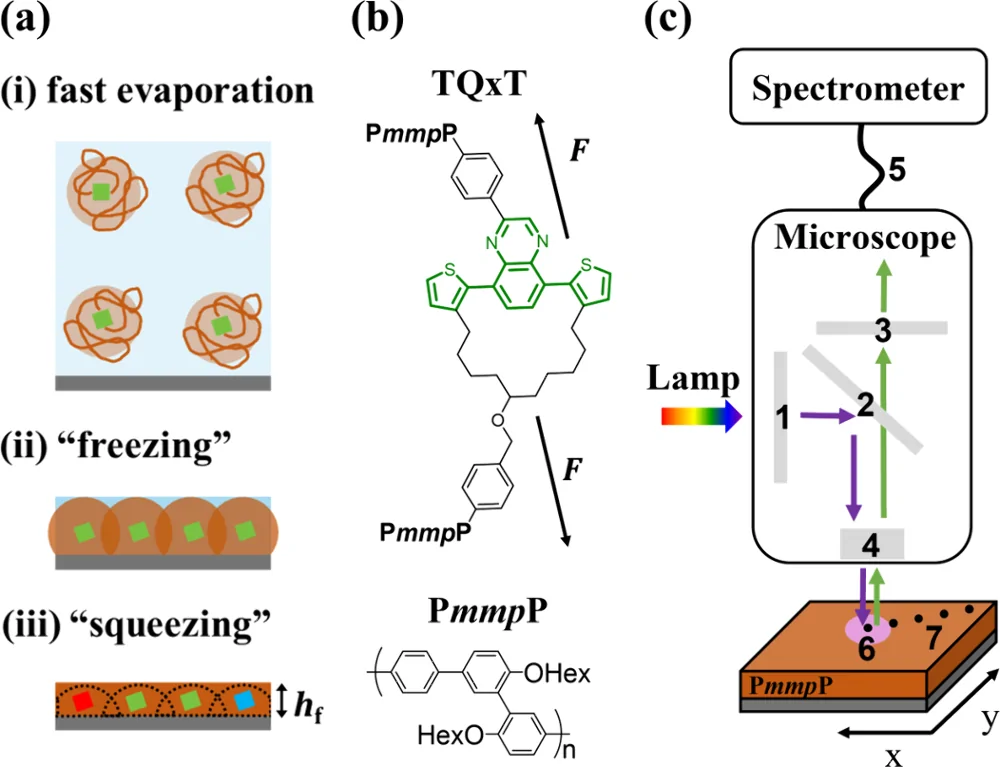
(a) Schematic illustration of characteristic stages of the spin coating process for the preparation of thin polymer films. (i) In the early stage, polymer chains are well separated. As the solvent evaporates, chains begin to overlap. (ii) Once the solution reaches the vitrification point, chain mobility becomes strongly suppressed (“freezing”). (iii) Deformation of the glassy film with significant content of solvent occurs predominantly in the thickness direction (“squeezing”). Eventually, a dry and uniform thin polymer film is obtained, with a final film thickness *h*_f_ determined mainly by the initial polymer concentration of the solution *c* and the spin speed ω. (b) Chemical structures of the mechanochromophore dithienylquinoxaline (TQxT) and the polymer poly­(*meta,meta,para*-phenylene) (P*mmp*P). The thiophene–quinoxaline–thiophene units undergo planarization under an applied force *F*, resulting in a monotonic increase of the photoluminescence (PL) wavelength. (c) Optical setup for PL measurements of thin polymer films. 1: Excitation filter. 2: Beamsplitter. 3: Emission filter. 4: Objective. 5: Optical glass fiber. 6: Illuminated area (violet). 7: Detected area (black dot within the violet area). To test the uniformity of the film, spectra were measured at various positions across the film indicated by black dots.

So far, residual stresses in spin-coated polymer films have been inferred mainly from macroscopic manifestations, such as dewetting^15,16^ and wrinkling instabilities,^17^ and have also been experimentally quantified.^18,19^ These stresses can strongly influence film stability, mechanical integrity, physical aging, and may cause failure in applications of thin polymer films.^20–22^ However, macroscopic approaches provide only spatially averaged information. Although recent studies have begun to probe residual stress at the molecular level,^23,24^ the variability of forces acting along individual polymer chains remains insufficiently understood.

Recently, mechanochromophores based on conformational changes of donor–acceptor–donor (DAD) compounds like dithienylquinoxaline (TQxT) were covalently incorporated into the backbone of glassy poly­(*meta,meta,para*-phenylene) (P*mmp*P) (Figure 1b),^25,26^ whose glass-transition temperature *T*_g_ is approximately 110 °C.^27^ Under an applied tensile force *F*, the dihedral angles within the DAD unit decrease, leading to planarization and enhanced conjugation. An increase in F results in a monotonic (approximately linear) shift Δλ_max_ of the position of the peak in the photoluminescence (PL) spectrum.^25^ This well-defined relation of *F* ∼ Δλ_max_ allows the mechanochromophores to be denoted as “optical springs”.

Here, we employed TQxT-P*mmp*P to optically monitor the forces acting along individual polymer chains in spin-coated films. In particular, we determined how these forces, their strength and variability depended on spin coating conditions. With such information, we aim to reveal how these molecular forces are related to the macroscopically observed residual stresses. To connect macroscopic residual stress with its molecular origin at the level of individual chains, we combined optical microscopy with optical spectroscopy. We accessed the differences between the acting forces via the shape of the PL spectra, reflecting the variations of the forces on a chain-to-chain basis, i.e., characterizing the distribution of preparation-induced molecular forces. With the quantification of the variability in force, we were also capable of identifying the corresponding preparation-induced fractions (relative number densities of groups of chains, i.e., force populations) of polymer chains experiencing a given force.

As a representative case, we used a film spin coated at 1000 rpm from a 10 mg/mL solution in CHCl_3_ with thickness *h*_f_ ≈ 150 nm. Several PL spectra were collected at distances from the rotation center of the substrate ranging from 0 to 4 mm (Figure 2a). Within experimental uncertainty, these spectra overlapped well, showing only negligible variations in intensity and shape and indicating that film thickness and optical response were uniform across the whole sample. Therefore, despite the presence of centrifugal forces during spin coating, the origin of residual stresses was not caused by the initial flow conditions during spin coating. Instead, consistent with previous observations,^11,28,29^ residual stresses were generated during the stage governed by solvent evaporation and ensuing vitrification. While stresses were uniform on the macroscopic scale, the spatial averaging across the detection area of ca. 50 μm^2^, inherent to our measurement approach, did not exclude variations on the molecular scale, where individual polymer chains could still experience different forces.

**Figure 2:**
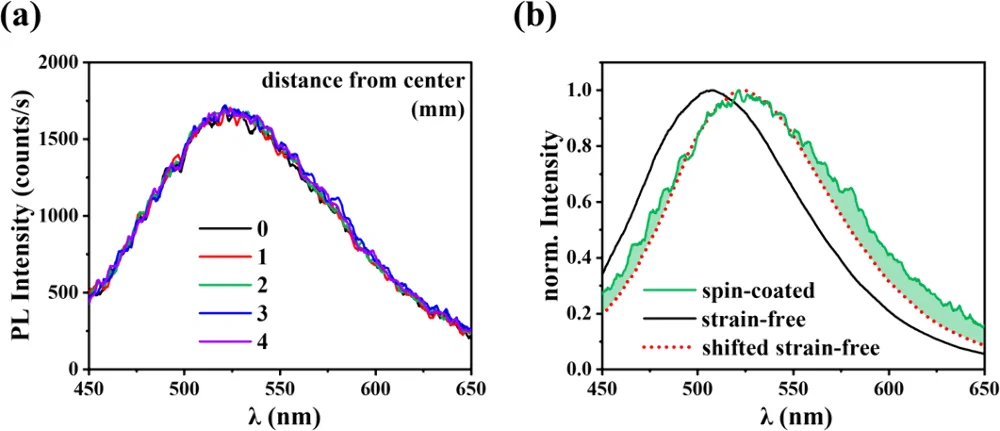
Characteristic PL spectra from a spin-coated film prepared from a 10 mg/mL solution at 1000 rpm. (a) Spectra collected at radial positions at 0 mm to 4 mm from the center are essentially identical, indicating lateral film homogeneity. (b) One spectrum (green line) arbitrarily chosen from the spectra shown in (a) was normalized and compared with a normalized PL spectrum (black line, denoted as “strain-free” spectrum) measured on an undeformed bulk specimen prepared by drop casting (without any applied force).^25^ The red dotted line represents the strain-free spectrum, which was shifted to higher wavelengths to match the peak position of the spin-coated spectrum. The shaded green area highlights the difference between the spin-coated spectrum and the shifted strain-free spectrum.

As a reference “strain-free” PL spectrum for a sample where no macroscopic deformations were imposed, we used the PL spectrum measured on an undeformed bulk specimen, prepared by drop casting (*h*_f_ ≈ 110 μm) under slow solvent evaporation conditions, as reported in ref 23. In Figure 2b, we compared this “strain-free” spectrum with one arbitrarily chosen spectrum from Figure 2a. Relative to the “strain-free” reference spectrum, the spectrum from the spin-coated film exhibited a clear red shift of λ_max_, indicating an increase in the tensile force acting along a significant fraction of polymer chains, i.e., confirming the presence of residual stresses of predominantly tensile character in the spin-coated film. For TQxT, it was previously shown that the full width at half-maximum (fwhm) decreased monotonically under an externally applied tensile strain.^25^ For a graphical comparison of the width of the spectra, the “strain-free” reference spectrum was horizontally shifted until the shifted λ_max_ coincided with the one from the spectrum of the spin-coated film. Intriguingly, the spectrum of the spin-coated film turned out to be noticeably broader on both sides, i.e., on the low wavelength (low strain) and the high wavelength (high strain) side of the spectrum. This observation demonstrated that spin coating induced not only a higher tensile force acting on a large number of mechano-chromophores, but also exhibited a broad distribution of populations of polymer chains experiencing lower and larger acting forces.

Taken together, our observations clearly showed that, even without deformation of the sample by applying an external force, residual stresses existed in the films prepared by spin coating. To further understand more precisely how processing conditions influenced these residual stresses, we systematically varied the spin speed ω and the concentration *c* of polymers in solution. In a first series of experiments, *c* was fixed at 10 mg/mL while ω was varied in the range from 1000 to 10000 rpm. As shown in Figure 3a for increasing ω, the normalized PL spectra progressively blue-shifted and became narrower, suggesting a reduction in the main tensile force as well as a narrowing of the distribution in the values of the forces experienced by the individual polymer chains. In the second series of experiments, ω was held constant at 4000 rpm while *c* was varied from 5 to 20 mg/mL. As shown in Figure 3b, the normalized spectra red-shifted and broadened with increasing concentration *c*, suggesting an increase in the main tensile force and a widening of the force distribution within the film. In both series, thicker films exhibited spectra which were more significantly red-shifted, suggesting higher tensile forces acting on the mechanochromophores. Moreover, by simple horizontal shifts, the spectra obtained under different preparation conditions did not perfectly superimpose, demonstrating that differences in the preparation conditions not only had an influence on λ_max_ (the main force) but were also reflected in variations in the distribution of the populations of chains experiencing various values of the force acting on them.

**Figure 3:**
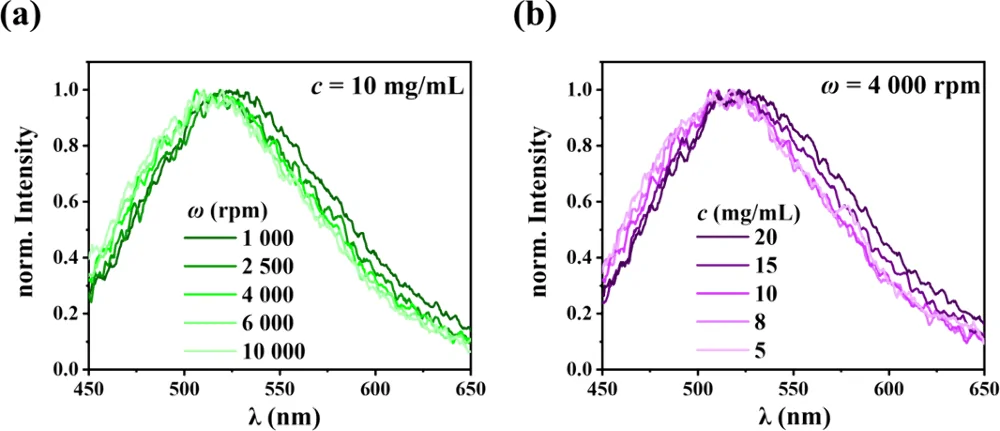
Influence of preparation conditions on normalized PL spectra from spin-coated films. (a) Films prepared from a *c* = 10 mg/mL CHCl_3_ solution at spin speed ω varied from 1000 to 10000 rpm. (b) Films prepared at ω = 4000 rpm from CHCl_3_ solutions with polymer concentration *c* varying from 5 to 20 mg/mL. The decrease in the shade of the color from dark to light indicates the corresponding decrease in film thickness imposed by the change in preparation conditions.

As an approach to combine and consistently compare our results from the two series of films based on variations in ω and *c*, respectively, we plotted λ_max_ as a function of the final thickness *h*_f_ of the dry film resulting from spin coating. As shown in Figure 4a, λ_max_ red-shifted with increasing *h*_f_, indicating that thicker films exhibited higher main residual stresses. This influence of spin coating conditions is consistent with some previous reports,^17,30,31^ while deviations from this behavior have also been reported.^16,32^ Clearly, the behavior of polymer films is not determined by film thickness alone,^10^ pointing to the role of processing-dependent parameters.

**Figure 4:**
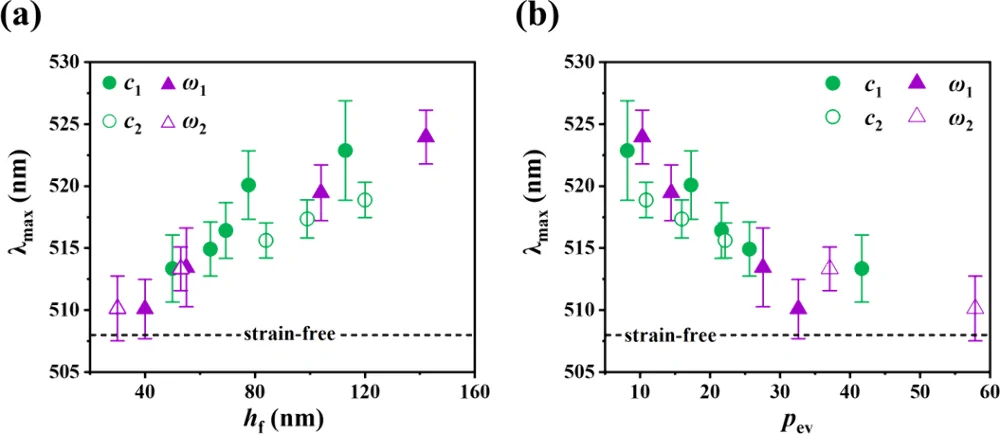
(a) Peak position λ_max_ versus *h*_f_. (b) λ_max_ versus the dimensionless preparation parameter *p*_ev_, defined as the ratio of a reference relaxation time τ_ref_ to the time *t*_ev_ required for complete removal of the solvent remaining at the point of vitrification of the solution film. Data are grouped by *c* (*c*_1_ = 10 mg/mL, *c*_2_ = 15 mg/mL) and ω (ω_1_ = 4000 rpm, ω_2_ = 8000 rpm). The black dashed lines indicate the peak position of the strain-free specimen.

Additionally, to verify that the observed changes in λ_max_ were independent of the analysis method, we calculated the first spectral moment ⟨*E*⟩, which probes the average emission energy of the spectrum for each sample (Figure S4). A similar trend as observed for λ_max_ (*h*_f_) was obtained for ⟨*E*⟩(*h*_f_), i.e., as *h*_f_ increased the values of ⟨*E*⟩ decreased, corresponding to an increase in the values of the mean force.

To account for this dependence on processing pathways, we employed the dimensionless preparation parameter *p*_ev_ proposed in a previous work.^10^ *p*_ev_ characterizes the competition between two key time scales during spin coating: the relaxation time of polymer segments under comparable (reference) conditions, τ_ref_, and the duration *t*_ev_ of the spin coating process between freezing-in of chain relaxations and complete evaporation of the solvent. During *t*_ev_, continued solvent loss induced a contraction in film thickness while the lateral dimensions were constrained by the substrate, contributing to the buildup of residual stresses. Accordingly, *p*_ev_ reflects the interplay between solvent-loss-induced stress generation and (incomplete) local relaxation processes. *p*_ev_ is defined as

1 $$p_{\mathrm{ev}}=\frac{\tau_{\mathrm{ref}}}{t_{\mathrm{ev}}}\approx \tau_{\mathrm{ref}}\cdot \Phi_{0}\cdot \frac{D}{h_{f}^{2}}\propto \frac{\omega^{0.6}}{c}$$

where Φ_0_ is the initial volume fraction of polymer in the solution, D is the relevant diffusion constant of the polymer. Thus, *p*_ev_ is jointly controlled by the spin speed ω and the solution concentration *c* (details are provided in the Supporting Information (SI): S4). Since neither the molecular weight nor the nature of the polymer was varied in this study, τ_ref_ was treated as constant. For simplicity, we have set τ_ref_ = 1 s.

As shown in Figure 4b, with increasing *p*_ev_, λ_max_ blue-shifted, indicating lower forces in films prepared at higher *p*_ev_. Increasing *p*_ev_ may not only reduce the time available for relaxation, but also shorten the time window during which solvent-loss-induced contraction of polymer chains can build up tensile forces. Even if the overall chain conformations in the glassy film were effectively frozen, as solvent evaporation continued, local segmental rearrangements would still be possible and could partially modify the local environment around the mechanochromophore. Such local segmental motion is consistent with the physical aging of glassy polymers, where nonequilibrium states try to relax toward equilibrium through local segmental rearrangements.^9^

However, this decrease of forces with increasing *p*_ev_ is opposite to that observed in dewetting experiments performed on PS films.^10^ Tentatively, we attribute this disagreement to different ways of probing the mechanical response in the two approaches. Dewetting experiments reflect the collective and averaged response of polymer chains in the film. By contrast, mechanochromophores report the sum of different local force states at the level of individual polymer segments. Similar differences in cross-scale comparisons have been reported for precision-engineered DNA hydrogels, where molecular force responses were linked to mesoscale and macroscopic nonlinear mechanics.^33^

To reveal how preparation conditions caused changes in the various populations of polymer chains experiencing different forces that are responsible for changes in the shape of PL spectra, we selected two characteristic regions of the spectrum. These selected windows correspond to polymer chains under comparatively low and high forces, respectively, which we labeled as “low-strain” and “high-strain” region in Figure 5a, corresponding to comparatively low and high forces acting along the polymer chains. Within each window, we approximated the normalized intensity *I*_norm_(λ) by a linear relation of the form *I*_norm_(λ) = (***a*** ± Δ ***a***) + ***b*** λ (see SI: S5 for details). Accordingly, our approach does not represent a quantitative reconstruction of the full distribution of the various populations of forces acting on the mechanochromophores, but mainly served as a rough approximation of the evolution of the spectra with preparation conditions. Of course, an accurate analysis requires to account for the variations in the shape of the spectra in a more profound and detailed way.

**Figure 5:**
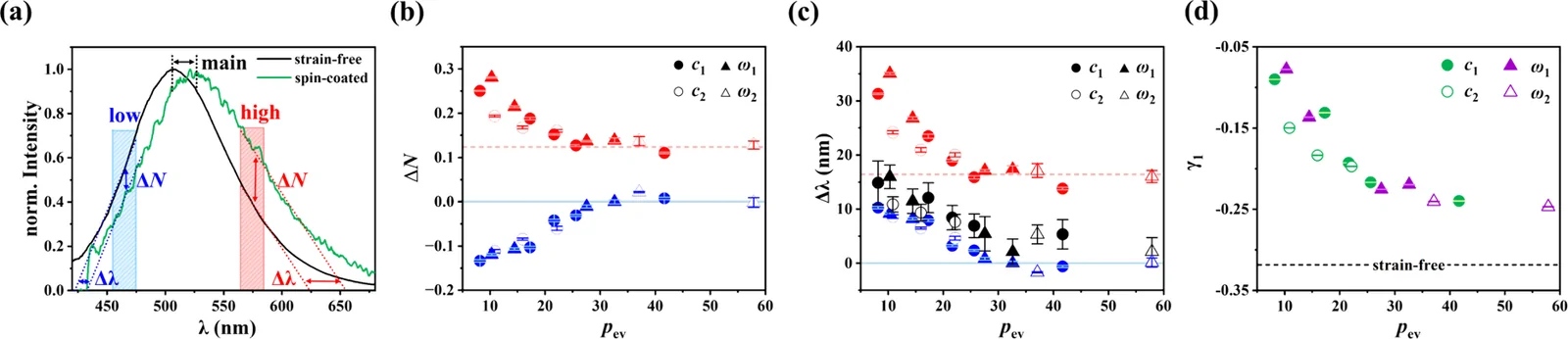
(a) Illustration of the approximate analysis of changes in the shape of PL spectra, using two 20 nm windows on the two sides around λ_max_, representing low-strain and high-strain regions, respectively. Within each window, the normalized PL intensity *I*_norm_ was approximated by a linear relation *I*_norm_(λ) = (***a*** ± Δ ***a***) + ***b*** λ, where ***b*** is fixed at constant values for the low- and high-strain regimes, respectively. Red and blue colors denote the high-strain and low-strain regions, respectively. (b) Values of Δ*N* = ***a*** – ***a***_strain–free_ for the two deformation regimes plotted against *p*_ev_. (c) Corresponding shifts of the wavelength Δλ = Δ*N*/***b*** plotted against *p*_ev_. The black symbols show the shift of λ_max_ relative to the strain-free specimen. (d) The third central moment γ_1_ (skewness) plotted against *p*_ev_. The black dashed line indicates the value of the strain-free specimen.

To quantify changes in the spectra, we determined the deviations from the strain-free reference spectrum. Derived from our linear relation for *I*_norm_(λ), we defined two representative parameters: Δ*N* = ***a*** – ***a***_strain–free_ and Δλ = – Δ*N*/***b***. Δ*N* reflects changes in the relative number of mechanochromophores contributing within the given spectral window. Converting Δ*N* into an effective shift in wavelength, i.e., into Δλ, allowed to express the response as a change in the corresponding force acting along this population of polymer chains. Such an analysis allowed to compare Δλ to the shift of the position of the maximum intensity of the spectrum, i.e., Δλ_max_, which represents the main force. The subscripts “low” and “high” refer to values extracted from the low- and high-strain regions, respectively.

Figure 5b shows, as a function of increasing *p*_ev_, that Δ*N*_low_ and Δ*N*_high_ evolved in opposite directions. Specifically, Δ*N*_low_ decreased, whereas Δ*N*_high_ increased, indicating that the respective populations of polymer chains changed differently with preparation conditions. At higher values of *p*_ev_, Δ*N*_low_ approached zero, indicating that mechanophores experiencing small deformations could reach a state close to that of the strain-free reference spectrum, i.e., a state where none of the polymer chains experienced weak forces. In contrast, Δ*N*_high_ remained finite for all samples. This persistent deviation from the strain-free sample indicated that a (small) fraction of polymer chains was always experiencing comparatively high forces.

As shown in Figure 5c, Δλ decreased with increasing *p*_ev_ for both the low-strain and high-strain regions. This change of Δλ­(*p*_ev_) is consistent with the shift observed for the main force acting along polymer chains represented by Δλ_max_ (*p*_ev_). As Δλ_max_ (*p*_ev_) decreased with *p*_ev_, inferring that the value of the main force acting on the mechanochromophores decreased, Δλ_low_(*p*_ev_) and Δλ_high_(*p*_ev_) also decreased with *p*_ev_ but with distinct differences. While both, Δλ_low_(*p*_ev_) and Δλ_high_(*p*_ev_), seemed to level off for the highest values of *p*_ev_, the differences to the reference spectrum were almost zero for Δλ_low_(*p*_ev_) but stayed finite for Δλ_high_(*p*_ev_). Our results show that preparation conditions do not uniformly change all populations of polymer chains experiencing different forces. Tentatively, we conclude that higher tensile forces were affected more strongly by preparation conditions characterized by the values of *p*_ev_.

Statistically, changes in spectral asymmetry can be characterized by the skewness γ_1_. Derived from the third central moment of the spectral distribution (see SI: S6), γ_1_ provides complementary information on changes in the populations of polymer chains experiencing comparatively low and high forces. As shown in Figure 5d, since all spectra remained unimodal, negative values of γ_1_ indicate a stronger extension of the distribution toward the low-energy, high-strain side of the spectrum. The results obtained from the skewness analysis are consistent with the spectral analysis above. For small *p*_ev_, γ_1_ was slightly negative, indicating that most polymer chains experienced force values comparatively close to the mean force. With increasing *p*_ev_, however, the skewness became progressively more negative. This indicates that more and more polymers experienced larger forces, increasing the high-strain tail of the distributions.

To conclude, we employed donor–acceptor–donor torsional springs as optical force probes at the molecular level to investigate residual stresses in spin-coated polymer thin films. By combining optical microscopy with spectroscopy, we were able to locally resolve the response of the optical springs via the corresponding photoluminescence (PL) spectra within an area of ca. 50 μm^2^. With respect to the reference strain-free state of an as-prepared and undeformed bulk specimen, all PL spectra from spin-coated films showed a clear shift of their maximum toward longer wavelengths, and a distinct and asymmetric broadening of their shape. From our experiments, we conclude that residual stresses cannot be described appropriately through the value of λ_max_ alone because individual chains experience forces over a wide range, which depend nonuniformly on preparation conditions. Unfortunately, a direct correlation between the collective response observed in dewetting experiments and the local response revealed by photoluminescence has not been established yet. Generally, our work revealed a link between processing pathways and corresponding nonequilibrium chain conformations in spin-coated thin films, which, in turn, is reflected in variations in forces acting along individual polymer chains. The presented experiments provide an informative approach for probing the molecular origin of preparation-induced residual stresses and offer new insight into how processing histories influence the forces acting along individual chains in spin-coated thin polymer films.

## Supplementary Material



## Notes

Experiments, data processing, force estimation, preparation parameter calculations, linear fitting, and spectral moment calculations (PDF)

## References

[1] Zhou H., Wang H., Niu H., Zhao Y., Xu Z., Lin T. (2017). A Waterborne Coating System for Preparing Robust, Self-healing, Superamphiphobic
Surfaces. Adv. Funct. Mater..

[2] He Z., Ma M., Lan X., Chen F., Wang K., Deng H., Zhang Q., Fu Q. (2011). Fabrication of a Transparent Superamphiphobic
Coating with Improved Stability. Soft Matter.

[3] Mitzi D. B., Kosbar L. L., Murray C. E., Copel M., Afzali A. (2004). High-Mobility
Ultrathin Semiconducting Films Prepared by Spin Coating. Nature.

[4] Zaidan K. M., Hussein H. F., Talib R. A., Hassan A. K. (2011). Synthesis and Characterization
of (Pani/n-Si)­Solar Cell. Energy Procedia.

[5] Dong Z., He Q., Shen D., Gong Z., Zhang D., Zhang W., Ono T., Jiang Y. (2023). Microfabrication of Functional Polyimide Films and
Microstructures for Flexible MEMS Applications. Microsyst. Nanoeng..

[6] Komikado T., Inoue A., Masuda K., Ando T., Umegaki S. (2007). Multi-Layered
Mirrors Fabricated by Spin-Coating Organic Polymers. Thin Solid Films.

[7] Tyona M. D. (2013). A Theoritical
Study on Spin Coating Technique. Adv. Mater.
Res..

[8] Birnie, D. P. Spin Coating Technique. In Sol-Gel Technologies for Glass Producers and Users; Aegerter, M. A. , Mennig, M. , Eds.; Springer US: 2004; pp 49–55.

[9] Chandran S., Reiter G. (2019). Segmental Rearrangements
Relax Stresses in Nonequilibrated
Polymer Films. ACS Macro Lett..

[10] Chandran S., Handa R., Kchaou M., Al Akhrass S., Semenov A. N., Reiter G. (2017). Time Allowed for Equilibration
Quantifies
the Preparation Induced Nonequilibrium Behavior of Polymer Films. ACS Macro Lett..

[11] Croll S. G. (1979). The Origin
of Residual Internal Stress in Solvent-cast Thermoplastic Coatings. J. Appl. Polym. Sci..

[12] Katsumata R., Ata S., Kuboyama K., Ougizawa T. (2013). Evaporation Rate Effect on Starting
Point of Shrinkage Stress Development during Drying Process in Solvent
Cast Polymer Film. J. Appl. Polym. Sci..

[13] Meyerhofer D. (1978). Characteristics
of Resist Films Produced by Spinning. J. Appl.
Phys..

[14] Hall D. B., Underhill P., Torkelson J. M. (1998). Spin Coating of Thin and Ultrathin
Polymer Films. Polym. Eng. Sci..

[15] Reiter G., Ramezani F., Baschnagel J. (2022). The Memory
of Thin Polymer Films
Generated by Spin Coating. Eur. Phys. J. E.

[16] Chowdhury M., Al Akhrass S., Ziebert F., Reiter G. (2017). Relaxing Nonequilibrated
Polymers in Thin Films at Temperatures Slightly above the Glass Transition. J. Polym. Sci., Part B: Polym. Phys..

[17] Chung J. Y., Chastek T. Q., Fasolka M. J., Ro H. W., Stafford C. M. (2009). Quantifying
Residual Stress in Nanoscale Thin Polymer Films *via* Surface Wrinkling. ACS Nano.

[18] Ramezani F., Baschnagel J., Reiter G. (2020). Translating Molecular Relaxations
in Non-Equilibrated Polymer Melts into Lifting Macroscopic Loads. Phys. Rev. Mater..

[19] Yang M. H., Hou S. Y., Chang Y. L., Yang A. C.-M. (2006). Molecular Recoiling
in Polymer Thin Film Dewetting. Phys. Rev. Lett..

[20] Reiter G. (1992). Dewetting
of Thin Polymer Films. Phys. Rev. Lett..

[21] Chowdhury M., Sheng X., Ziebert F., Yang A. C.-M., Sepe A., Steiner U., Reiter G. (2016). Intrinsic Stresses in Thin Glassy
Polymer Films Revealed by Crack Formation. Macromolecules.

[22] Raegen A., Chowdhury M., Calers C., Schmatulla A., Steiner U., Reiter G. (2010). Aging of Thin Polymer Films Cast
from a Near-Theta Solvent. Phys. Rev. Lett..

[23] Ju J., Cheng B., Jiang Z., Yang J., Zhao J. (2025). Correlated
Molecular Motion during Release of Residual Stress in Polymer Glassy
Films. J. Phys. Chem. B.

[24] Janissen R., Filonenko G. A. (2022). Mechanochemistry
of Spiropyran under Internal Stresses
of a Glassy Polymer. J. Am. Chem. Soc..

[25] Hertel R., Maftuhin W., Walter M., Sommer M. (2022). Conformer Ring Flip
Enhances Mechanochromic Performance of *Ansa* -Donor–Acceptor–Donor
Mechanochromic Torsional Springs. J. Am. Chem.
Soc..

[26] Hertel R., Raisch M., Walter M., Reiter G., Sommer M. (2024). Mechanistically
Different Mechanochromophores Enable Calibration and Validation of
Molecular Forces in Glassy Polymers and Elastomeric Networks. Angew. Chem., Int. Ed..

[27] Kempe F., Brügner O., Buchheit H., Momm S. N., Riehle F., Hameury S., Walter M., Sommer M. (2018). A Simply Synthesized,
Tough Polyarylene with Transient Mechanochromic Response. Angew. Chem..

[28] Reiter G., De Gennes P. G. (2001). Spin-Cast,
Thin, Glassy Polymer Films: Highly Metastable
Forms of Matter. Eur. Phys. J. E.

[29] Francis L. F., Mccormick A. V., Vaessen D. M., Payne J. A. (2002). Development and
Measurement of Stress in Polymer Coatings. Journal
of Materials Science.

[30] Zhang H., Zhang L. (2015). Residual Stress in
Spin-Cast Polyurethane Thin Films. Appl. Phys.
Lett..

[31] Ree M., Chu C.-W., Goldberg M. J. (1994). Influences of Chain Rigidity, in-Plane
Orientation, and Thickness on Residual Stress of Polymer Films. J. Appl. Phys..

[32] Damman P., Gabriele S., Coppée S., Desprez S., Villers D., Vilmin T., Raphaël E., Hamieh M., Akhrass S. A., Reiter G. (2007). Relaxation of Residual
Stress and Reentanglement of
Polymers in Spin-Coated Films. Phys. Rev. Lett..

[33] Lallemang M., Akintayo C. O., Wenzel C., Chen W., Sielaff L., Ripp A., Jessen H. J., Balzer B. N., Walther A., Hugel T. (2023). Hierarchical Mechanical Transduction of Precision-Engineered DNA
Hydrogels with Sacrificial Bonds. ACS Appl.
Mater. Interfaces.

