# Supplementary material for: Determining Preparation-Induced Differences in Local Forces Acting along Polymer Chains in Spin-Coated Films
Source: ACS Macro Lett. 2026 Jul 24;15(8):1157–62. doi: 10.1021/acsmacrolett.6c00311 (PMC13492362; doi:10.1021/acsmacrolett.6c00311)
Supplement: Supplementary file 1 [file mz6c00311_si_001.pdf]

Supporting Information

for

Determining Preparation-Induced Differences in Local Forces Acting along Polymer Chains in Spin-Coated Films

Meirui Fu[1], Raphael Hertel[2], Bence Dajka[3], Michael Walter[3], Michael Sommer[2], and Günter Reiter*[1]

[1]Institute of Physics, University of Freiburg, Freiburg 79104, Germany

[2]Institute for Chemistry, Chemnitz University of Technology, Chemnitz 09111, Germany

[3]FIT Freiburg Centre for Interactive Materials and Bioinspired Technologies, University of Freiburg, Freiburg 79110, Germany

S1. Experiments

Poly(*meta*,*meta*,*para*-phenylene) (P*mmp*P) (M_n = 32 kg/mol, M_w = 148 kg/mol, containing 3 mol% of dithienylquinoxaline (TQxT) units) was spin coated onto silicon substrates (1 cm ×1 cm) from chloroform ($CHCl_3$) solutions with concentrations between 5 to 20 mg/mL. The spin speed was varied between 1000 rpm and 10 000 rpm.

Photoluminescence (PL) spectra were collected directly from the spin coated films using a Zeiss Axio 100 microscope equipped with a 50× objective and coupled to a spectrometer via a 400 μm optical fiber. The mechanochromophores were excited via an HBO100 mercury lamp, which had

high radiant power in the near-UV range and was therefore well-suited for PL measurements on thin films requiring sufficient photon flux from small detection areas. The AHF filter set 26-000 was employed, consisting of an excitation filter transmitting 360–390 nm, a dichroic beam splitter with a cut-on wavelength of 400 nm, and a long-pass emission filter transmitting wavelengths above 435 nm. The excitation filter selected a wavelength range where only TQxT exhibited detectable absorbance, while absorbance and emission from the polymer backbone was not contributing significantly[1]. The dichroic beam splitter and the emission filter, in the direction from the sample to the spectrometer, efficiently suppressed most unwanted photons (e.g., from the excitation light source or stray light), ensuring that the detected signal originated from photons emitted from the mechanochromophores. The combination of a 50× objective and a 400 µm optical fiber provided a favorable balance between signal quality (high signal-to-noise ratio) and spatial resolution of different locations on the sample. With our set-up, we achieved an effective detection area of approximately 50 µm². Thus, we were able to perform PL measurements of well-defined regions chosen at various positions across the whole film. All PL spectra were acquired with an integration time of 1 s.

The final thickness h_f of the dry film was determined by atomic force microscopy (AFM). To this end, we generated a scratch in the polymer film and measured the step height between the polymer film surface and the exposed silicon substrate.

S2. Data processing

Calibration. To correct for the spectral response of the detection system, each measured spectrum was calibrated using a certified halogen calibration lamp (AvaLight-HAL-CAL-Mini, 350 –1095 nm). For each wavelength λ, the measured intensity $I_\mathrm{meas}(\lambda)$ was multiplied by a wavelength-dependent calibration factor $C(\lambda) = \frac{I_\mathrm{lamp}^\mathrm{ref}(\lambda)}{I_\mathrm{lamp}^\mathrm{meas}(\lambda)}$, where $I_\mathrm{lamp}^\mathrm{ref}(\lambda)$ refers to the certified, normalized reference spectrum supplied by the manufacturer, and $I_\mathrm{lamp}^\mathrm{meas}(\lambda)$ refers to the corresponding measured normalized spectrum obtained with the same optical setup. The calibrated spectrum used in all subsequent analyses is therefore

$$I_\mathrm{cal}(\lambda) = I_\mathrm{meas}(\lambda) \cdot \mathrm{C}(\lambda). \tag{1}$$

Smoothing. To further improve the signal-to-noise ratio without distorting spectral features, the calibrated spectra were smoothed using a Savitzky–Golay filter with a third-order polynomial and a window size of 13 data points.

Normalization. The normalized spectra were obtained from the smoothed spectra by dividing by the maximum intensity.

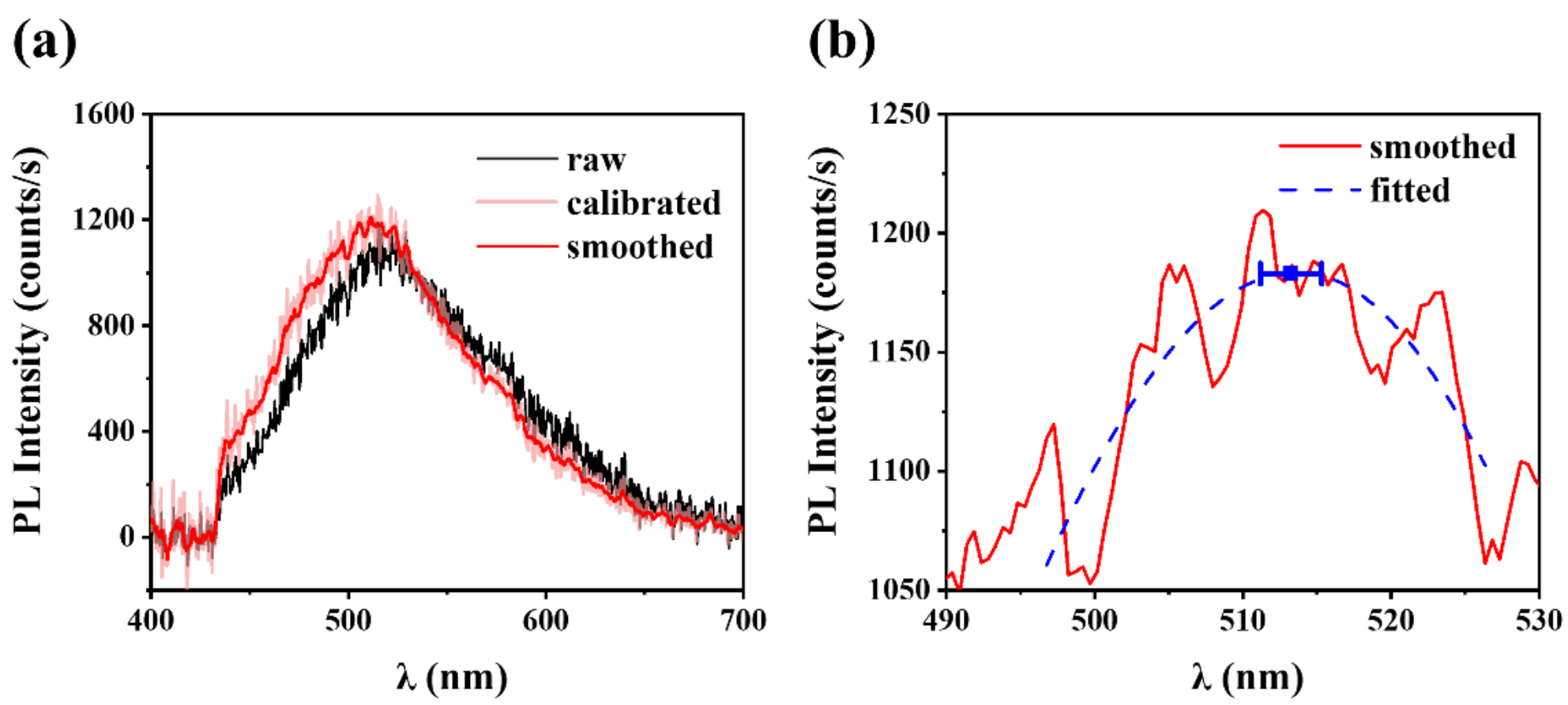

Figure S1. (a) Representative PL spectrum showing the **measured** raw data, the **calibrated** spectrum after correction for the spectral response of the detection system, and the **smoothed** spectrum. (b) Comparison between the smoothed spectrum and the fitted spectrum obtained using an asymmetric Gaussian function for a narrow wavelength range around the maximum peak.

Peak fitting. The peak position $\lambda_{\max}$ was determined by fitting the smoothed spectrum in the vicinity of the maximum using a composite Gaussian function. First, an approximate peak position was identified as the wavelength corresponding to the maximum intensity of the measured spectrum. A fitting window of ±15 nm around this position was then selected. Within this window, the spectral shape was fitted using an asymmetric Gaussian function composed of two parts of the form

$$I(\lambda) = A \exp\left[-\frac{1}{2}\left(\frac{\lambda - \lambda_0}{\sigma}\right)^2\right] \tag{2}$$

where different widths, $\sigma = \sigma_L$ or σ_R, were used for $\lambda < \lambda_0$ and $\lambda > \lambda_0$, respectively. The fitting parameters were the amplitude A, the peak position λ_0, and the left and right widths σ_L and σ_R.

The fitted peak position λ_0 was taken as λ_{max}. The uncertainty in the value of λ_{max} was estimated from the covariance matrix of the fit and reported as a 95% confidence interval.

S3. Force estimation

Previous computational work showed that the change in the experimentally observed PL wavelength of TQxT is governed by two mechanically distinct contributions, giving rise to two response regimes[1,2]. In regime I, at relatively small forces, various conformational rearrangements occur, including flipping of the aliphatic bridge and changes in the thiophene orientation. In regime II, at larger forces, progressive force-induced planarization of the π-system produces a further red shift of the PL spectrum. For forces up to approximately 1 nN in this second regime II, the planarization-induced PL shift was reported to scale linearly with force, with a proportionality factor of approximately 21 nm/nN[1,2]. In particular, ring flipping was predicted to occur at forces of up to approximately 27 pN[1], corresponding to an emission wavelength of about 525 nm[1], before the onset of force-induced planarization will take place at higher forces. Based on this established calibration, the values of λ_{max} measured for our samples correspond to an estimated force scale of approximately 0–265 pN, as shown by the right axis in Figure S2(a).

However, the measured PL spectrum is the sum of photons emitted from TQxT units experiencing different local forces, rather than the response to an average (equal) force acting on all segments along an entire polymer chain. Therefore, λ_{max} represents the main spectral response of the ensemble and cannot be assigned directly to one unique force acting on all chains or chain segments. The difficulty of applying a single force calibration to the ensemble PL spectrum is illustrated schematically in Figure S2(b). Different populations may fall into different mechanical response regimes and, in principle, the corresponding wavelength shifts should therefore be evaluated using the appropriate regime-specific calibrations. Applying a single calibration to an overall shift may not be adequate, particularly when the force distribution is broad. Analysis of selected regions may instead provide information about relative changes in different populations. However, assigning absolute forces would require separation of the individual spectral contributions and identification of the applicable response regime for each contribution (force population). Moreover, conversion of the spectral response into a macroscopic residual stress would require an additional model relating the

distribution of local forces along the polymer chains to the bulk mechanical response. It also remains unclear how the local forces sensed by the mechanochromophores are related to residual stresses measured on macroscopic length and time scales.

Accordingly, the force values estimated here from $\lambda_{\max}$ should be interpreted only as an approximate, literature-calibrated force scale rather and not as a unique or ensemble-averaged force.

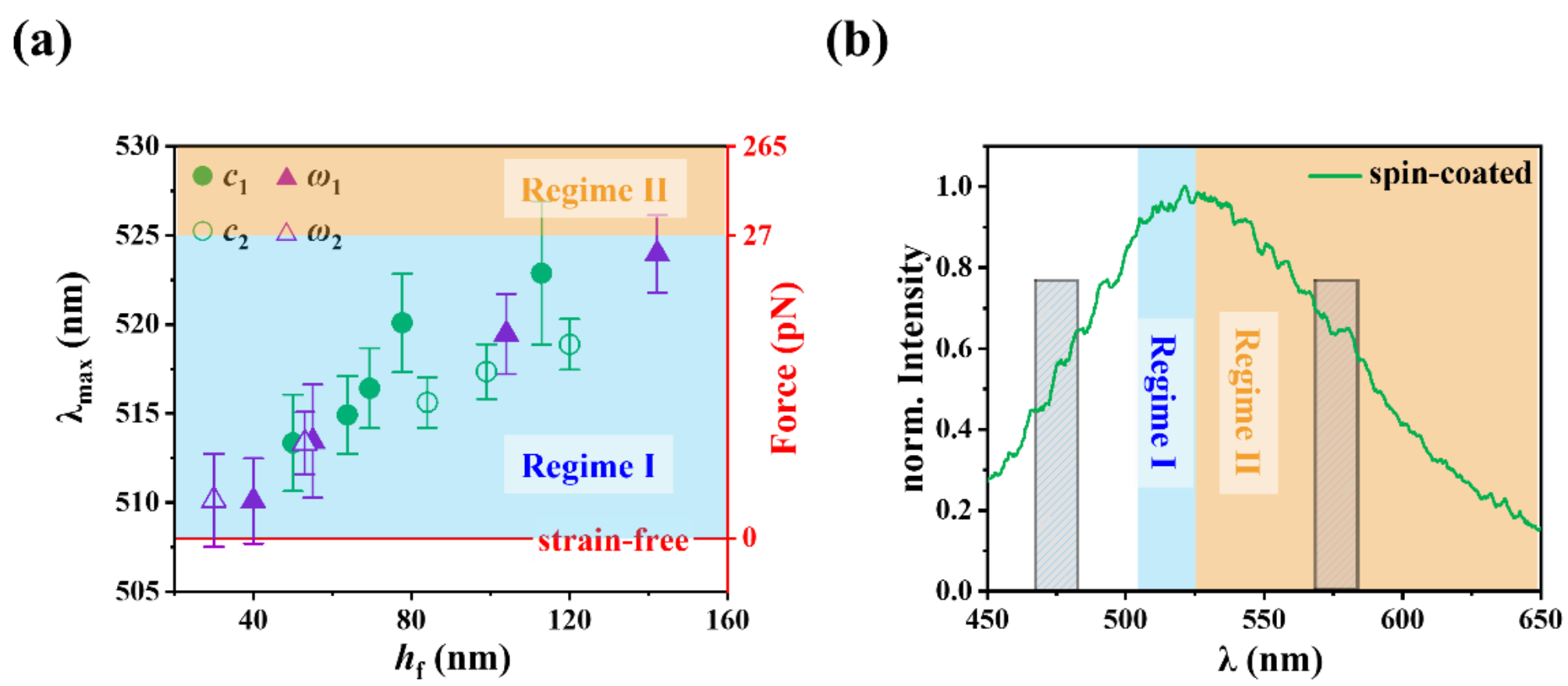

Figure S2. (a) Peak position $\lambda_{\max}$, as a function of the final film thickness h_{f}. The corresponding force scale estimated from the literature-established calibration is shown on the right axis, reflecting regime I(blue) from 0 pN to 27 pN and regime II(orange), from 27 pN to 265 pN. Please note that the force axis is not represented on a linear scale. (b) Representative normalized PL spectrum. The shaded windows schematically represent different populations that fall into different mechanical response regimes and therefore require regime-specific calibrations for force estimation.

S4. Preparation parameter p_{ev}

At early stages of spin coating, when the polymer concentration in the spread solution film is still dilute or semi-dilute, individual polymer chains are swollen by the solvent, often well separated, and relax fast. As the solvent continuously evaporates, the polymer concentration increases and surpasses the overlap concentration, with polymer chains interpenetrating and entangling partially. As a consequence, the relaxation time of polymer chains increases rapidly during the late stages of

solvent evaporation. Eventually, the time required for evaporation of the remaining solvent in the film becomes shorter than the chain relaxation time. The film thus enters a vitrification regime with strongly suppressed segmental mobility. This behavior can be rationalized by considering the competition between two characteristic time scales: a characteristic, concentration dependent relaxation time τ_{ref} describing the equilibration of polymer chains under comparable (reference) conditions, and the time t_{ev} corresponding to the time required for complete removal of the (remaining) solvent. When t_{ev} becomes shorter than τ_{ref}, polymer chains become trapped in nonequilibrium conformations.

Following Ref. [3], the time t_{ev} can be expressed as

$$t_{\mathrm{ev}} \approx \frac{h_{\mathrm{f}}^2}{\Phi_0 \cdot D} \tag{3}$$

where Φ_0 is the initial volume fraction of polymer in the solution, D is the relevant diffusion constant of the polymer.

Based on Eq. (3), we define the preparation parameter p_{ev} as

$$p_{\mathrm{ev}} = \frac{\tau_{\mathrm{ref}}}{t_{\mathrm{ev}}} \approx \tau_{\mathrm{ref}} \cdot \Phi_0 \cdot \frac{D}{h_{\mathrm{f}}^2}. \tag{4}$$

Since neither the chemical nature nor the molecular weight of the polymer was varied in this study, D and τ_{ref} were treated as constant. Φ_0 depends on the polymer concentration c as $\Phi_0 = c/\rho_{\mathrm{p}}$, with ρ_{p} being the density of polymer P*mmp*P ($\rho_p \approx 0.96\ g\ cm^{-3}$ [4]).

Typically, h_{f} follows the scaling relation $h_f \propto c^a \cdot \omega^{-b}$ [5]. In the present experiments, fitting the measured film thicknesses yielded $a \approx 1$ and $b \approx 0.3$ (Figure S3).

Consequently, p_{ev} was effectively governed by the spin speed ω and the polymer concentration c

$$p_{\mathrm{ev}} \propto \frac{\omega^{0.6}}{c} \tag{5}$$

Thus, the preparation conditions were represented by p_{ev}.

For the estimation of p_{ev}, we used $D = 10^{-7}\ \mathrm{cm}^2/\mathrm{s}$ [6,7] and $\tau_{\mathrm{ref}} = 1$ s.

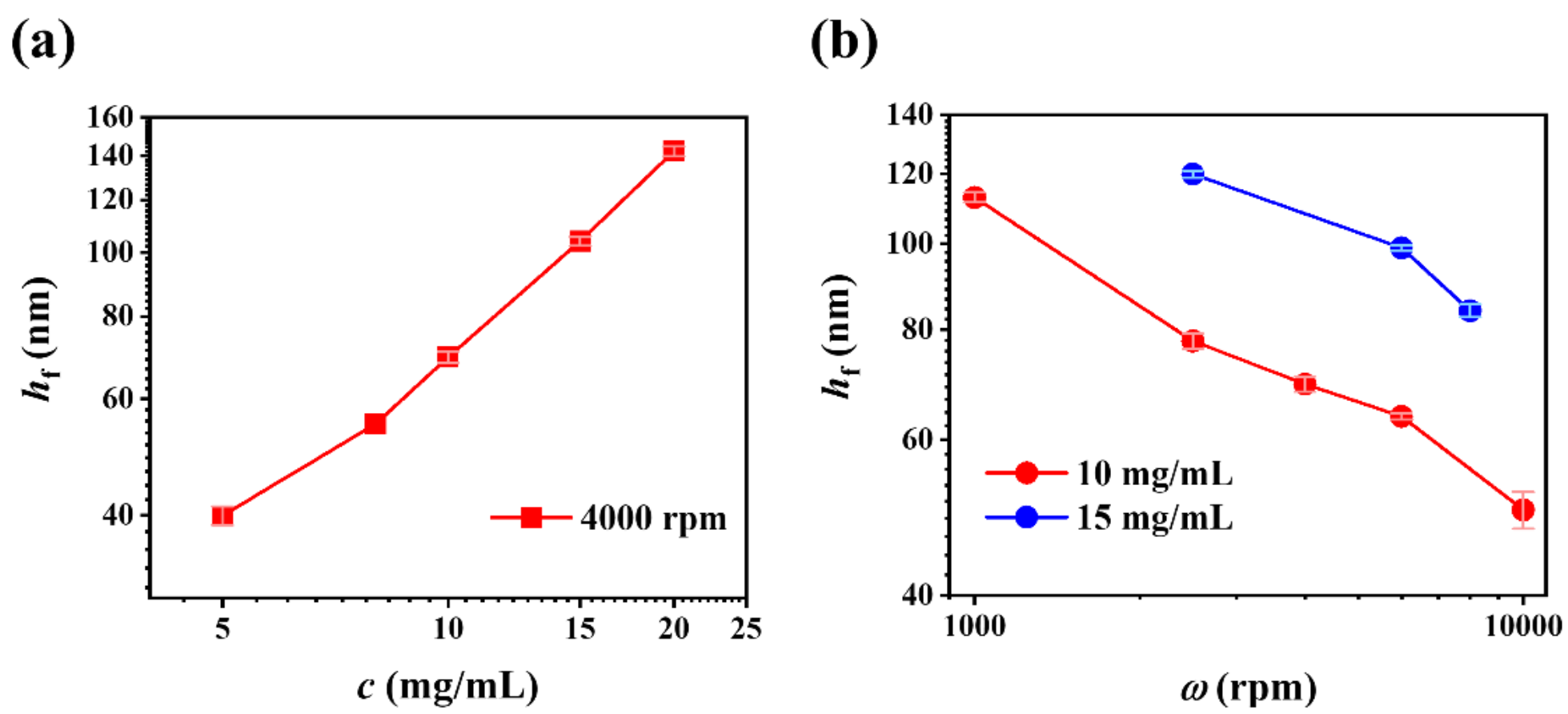

Figure S3. (a) h_{f} versus c. We observed $h_{\mathrm{f}} \sim c^{a}$, where $a \approx 1$. (b) h_{f} versus ω. We observed $h_{\mathrm{f}} \sim \omega^{-b}$, where $b \approx 0.3$.

S5. Linear fitting

We chose two narrow 20 nm wide windows approximately at the half-maximum positions on either side of λ_{max}, where the PL intensity exhibited the steepest variation with wavelength. Within each window, we approximated the normalized intensity $I_{\mathrm{norm}}(\lambda)$ by a linear relation of the form $I_{\mathrm{norm}}(\lambda) = (\boldsymbol{a} \pm \Delta\boldsymbol{a}) + \boldsymbol{b}\lambda$. For a robust comparison of spectra obtained from films prepared under different conditions, we fixed the value of the slopes $\boldsymbol{b}$. To account for the asymmetry of the spectra, we have chosen different constant values of $\boldsymbol{b}$ for the "low-strain" and "high-strain" regions, respectively. Even when we fitted the values of the slopes $\boldsymbol{b}$, the results consistently did not differ much for both the bulk reference and spin coated films. For our comparison of results obtained for different preparation conditions, we used **b** = -0.008 or 0.013 nm^{-1} for the "low-strain" and "high-strain" regions, respectively. The reduction in fitting parameters allowed to focus on the relative changes in PL intensity at the chosen positions at approximately the half-maximum PL intensity on either side of λ_{max}.

S6. Spectral moment calculations

To ensure the validity of the obtained results, a statistically robust method was used in concert with the estimation of the peak position. The PL spectrum was treated analogously to a probability density distribution after normalization by the integrated intensity. Within this framework, the *n*-th spectral moment is defined as

$$\mu_n = \int E^n \cdot I(E)\ dE \tag{8}$$

where $I(E)$ denotes the measured PL intensity at the photon energy E, and μ_n represents the energy-weighted integral of order *n*. By averaging over the whole spectra, the spectral moments describe central values, characterizing the whole distribution rather than only the peak position.

The first central moment μ_1,

$$\mu_1 = \langle E \rangle = \frac{\int E\ \cdot I(E)\, dE}{\int I(E)\ dE} \tag{9}$$

corresponds to the average emission energy of the spectrum and can be interpreted as the mean energy of the emitted photons. In contrast to the spectral maximum, this quantity reflects the weighted contribution of the entire spectrum and is therefore less sensitive to local noise or small irregularities in the spectra.

The second central moment μ_2,

$$\mu_2 = \sigma^2 = \langle (E - \langle E \rangle)^2 \rangle = \frac{\int (E - \langle E \rangle)^2\ I(E)\, dE}{\int I(E)\ dE} \tag{10}$$

describes the variance of the spectral distribution around the average emission energy. The square root of this quantity, σ, provides a measure of the spectral width and thus reflects the energetic broadening of the distribution. As shown in Figure S4, compared to the strain-free reference spectrum, only minor variations in the spectral width were observed over the investigated p_{ev} range, indicating that the overall energetic broadening of the spectra remained largely unchanged despite the different preparation conditions.

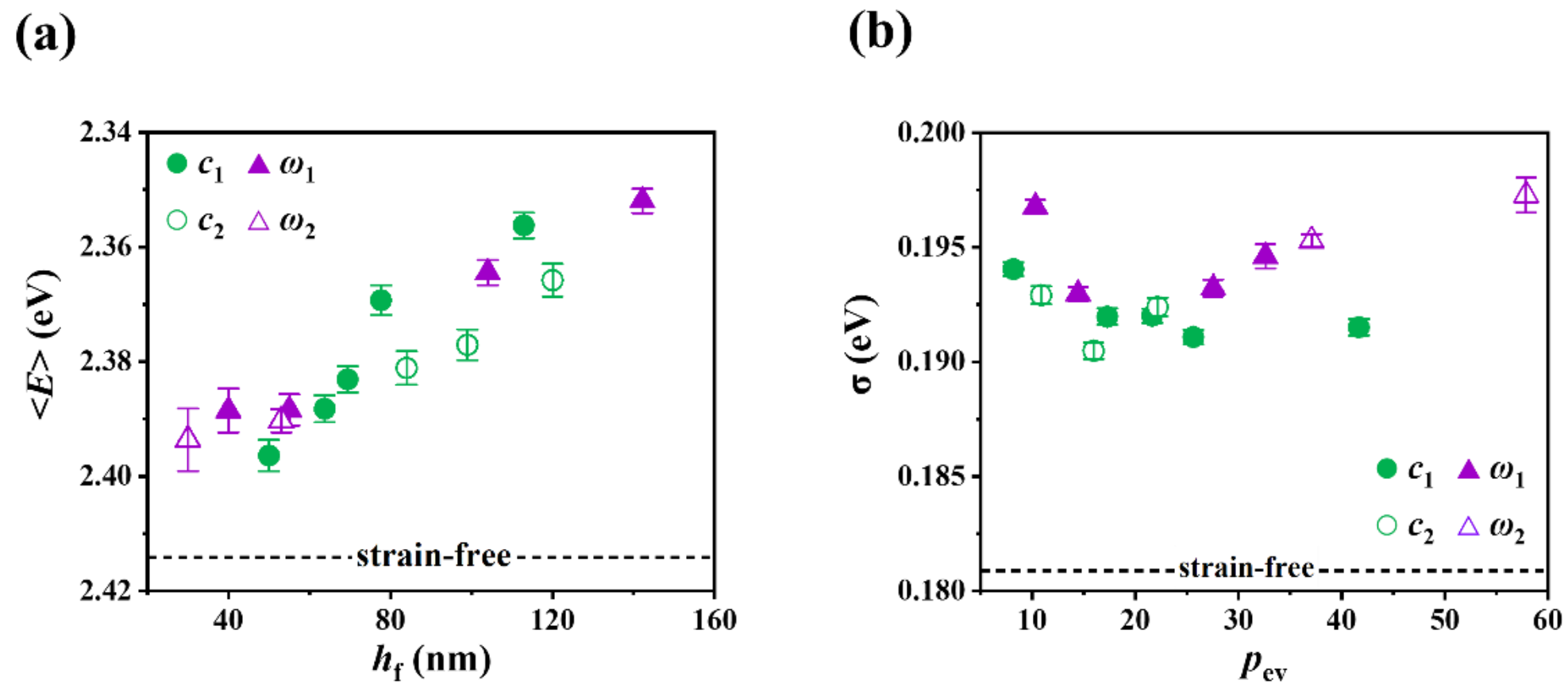

Figure S4. (a) The first central moment $\langle E\rangle$ versus h_f similar to Fig.4 in the main text. (b) The spectral width σ, obtained from the second central moment of the PL spectra, plotted as a function of p_ev.

The third standardized moment μ_3, also denoted as γ_1,

$$\mu_3 = \gamma_1 = \left\langle\left(\frac{E-\langle E\rangle}{\sigma}\right)^3\right\rangle = \frac{1}{\sigma^3}\frac{\int (E-\langle E\rangle)^3\ I(E)\,dE}{\int I(E)\ dE} \tag{11}$$

corresponds to the skewness of the spectrum and quantifies the asymmetry of the intensity distribution relative to the average emission energy. Positive or negative values of γ_1 indicate enhanced spectral weight on the high- or low-energy side of the spectrum, respectively.

The uncertainties of the experimentally determined moments were estimated using a Monte Carlo approach: artificial noise, based on the experimental signal fluctuations, was added repeatedly to the measured spectra. The corresponding distributions of the calculated moments were used to estimate the statistical uncertainties.

References

(1) Hertel, R.; Maftuhin, W.; Walter, M.; Sommer, M. Conformer Ring Flip Enhances Mechanochromic Performance of *Ansa* -Donor–Acceptor–Donor Mechanochromic Torsional Springs. *J. Am. Chem. Soc.* **2022**, *144* (48), 21897–21907.

(2) Hertel, R.; Raisch, M.; Walter, M.; Reiter, G.; Sommer, M. Mechanistically Different Mechanochromophores Enable Calibration and Validation of Molecular Forces in Glassy Polymers and Elastomeric Networks. *Angew. Chem. Int. Ed.* **2024**, e202409369.

(3) Chandran, S.; Handa, R.; Kchaou, M.; Al Akhrass, S.; Semenov, A. N.; Reiter, G. Time Allowed for Equilibration Quantifies the Preparation Induced Nonequilibrium Behavior of Polymer Films. *ACS Macro Lett.* **2017**, *6* (11), 1296–1300.

(4) Fenton, A. M.; Xie, R.; Aplan, M. P.; Lee, Y.; Gill, M. G.; Fair, R.; Kempe, F.; Sommer, M.; Snyder, C. R.; Gomez, E. D.; Colby, R. H. Predicting the Plateau Modulus from Molecular Parameters of Conjugated Polymers. *ACS Cent. Sci.* **2022**, *8* (2), 268–274.

(5) Lawrence, C. J. The Mechanics of Spin Coating of Polymer Films. *Phys. Fluids* **1988**, *31* (10), 2786–2795.

(6) Danglad-Flores, J.; Eickelmann, S.; Riegler, H. Evaporation Behavior of a Thinning Liquid Film in a Spin Coating Setup: Comparison between Calculation and Experiment. *Eng. Rep.* **2021**, *3* (9), e12390.

(7) Rauch, J.; Köhler, W. Diffusion and Thermal Diffusion of Semidilute to Concentrated Solutions of Polystyrene in Toluene in the Vicinity of the Glass Transition. *Phys. Rev. Lett.* **2002**, *88* (18), 185901.
